# Hypertrophic Pyloric Stenosis in Dizygotic Twins: Case Report

**DOI:** 10.7759/cureus.20350

**Published:** 2021-12-11

**Authors:** Ishan Poudel

**Affiliations:** 1 Pediatrics, Woodhull Medical Center, Brooklyn, USA

**Keywords:** pylorus, infantile hypertrophic pyloric stenosis, dizygotic twins, ihps, hypertrophic pyloric stenosis

## Abstract

Infantile hypertrophic pyloric stenosis (IHPS) is a disorder encountered in infancy that is caused by hypertrophy of the musculature of the pylorus of the stomach. It may present as partial or complete gastric outlet obstruction. Multiple previous literatures have discussed the incidences and variability in the presentation of IHPS. However, there are very few reports of IHPS occurring in dizygotic twins, especially dizygotic twins of different sexes. Here we present a very rare case of dizygotic twins (a male and a female) affected with IHPS. With this study, we aim to identify the factors that lead to variability in severity and onset of symptoms in dizygotic twins of the opposite sex. We also aim to pay special attention to the etiology and mechanism of development of IHPS in dizygotic twins of the opposite sex.

## Introduction

The incidence of hypertrophic pyloric stenosis among infants in the US is one to three per 1000 infants. It is more commonly identified in whites of European ancestry, is less common among black infants and is rarely seen in Asian infants. It is four to six times more common in males than in females [1]. A large population registry data from Denmark evaluated for the familial association in pyloric stenosis and identified a 200-fold increase in risk among monozygotic twins and only a 20-fold increase in risk among dizygotic twins compared to the general population without known affected relatives [2]. Here we report a rare case of dizygotic twins of the opposite sex who were exclusively formula-fed presenting with infantile hypertrophic pyloric stenosis (IHPS) at different periods of life. We reviewed the literature focusing on whether environmental factors play a role in variability of the duration of symptom appearance.

## Case presentation

Dizygotic twins, 'Twin A' and 'Twin B' of opposite sex born in our facility at 36+1 weeks of gestation to a 24-year-old G3P1113 female with gestational diabetes mellitus well controlled on diet and positive Group B Streptococcus (GBS) culture adequately treated with Penicillin G, presented to our hospital network's emergency department at two and four weeks of life respectively with non-bilious vomiting after each feeding.

Twin A, the male infant born with weight of 3025g, length of 48cm and head circumference of 34cm who cried immediately after birth and passed meconium within the first 24 hours, was assigned a Ballard score of 37 weeks at birth. The infant had an uneventful nursery course with no history of jaundice during the nursery stay. The child was exclusively formula-fed and no history of IHPS could be elicited on either the paternal or maternal side of the family. He presented on the 19th day of life with non-bilious, non-bloody vomiting for 24 hours after every feed. The amount of wet and soiled diapers decreased with the onset of vomiting with no change in color or consistency of the stool. The weight on the day of the presentation was 3215g. Physical examination revealed active infants with good suck and cry, soft anterior fontanelle with adequate Moro's reflex, rooting reflex and capillary refill time of less than two seconds. There was no palpable mass on the abdominal examination and no visible peristalsis. The infant was fed 2 oz of formula in the emergency department and was observed to have two episodes of projectile vomiting within 10 minutes post-feed with expulsion amount almost equal to the total amount of ingested formula. There was no history of exposure to coronavirus disease 2019 (COVID-19) contacts.

Twin B, the female with a birth weight of 2191g with weight discordance of 20% to the first twin, length of 45 cm, 36 weeks by Ballard score, had Apgar scores of one, three, and nine at one minute, five minutes and nine minutes respectively. The infant received non-invasive positive pressure ventilation for two minutes after birth for poor respiratory effort and heart rate of less than 100, after two minutes of life patient received 30 minutes of positive end expiratory pressure (PEEP) at 5 cm of H_2_O for the presence of nasal flaring, retractions, and poor respiratory effort. Infant was observed for transient tachypnea of newborn (TTN) for two hours with no oxygen requirement and oxygen saturation of 95%. The infant passed meconium within 24 hours of birth with no concerns for neonatal jaundice during the neonatal course.

She presented on the fourth week of life with non-bilious, non-bloody, large-volume vomiting for two days after every feed with an unchanged number and appearance of wet and soiled diapers. The weight on the day of the presentation was 2600g. Physical examination revealed active infants with good suck and cry, soft anterior fontanelle with adequate Moro's reflex, rooting reflex and capillary refill time of less than two seconds, and normal skin pinch return time. Abdominal examination was significant for a fixed small palpable mass on the left upper quadrant with no visible peristalsis. There was no history of exposure to COVID-19 contacts. Patient presented with a positive history of IHPS in the twin so investigations were performed.

Investigations

Twin A

**Figure 1:**
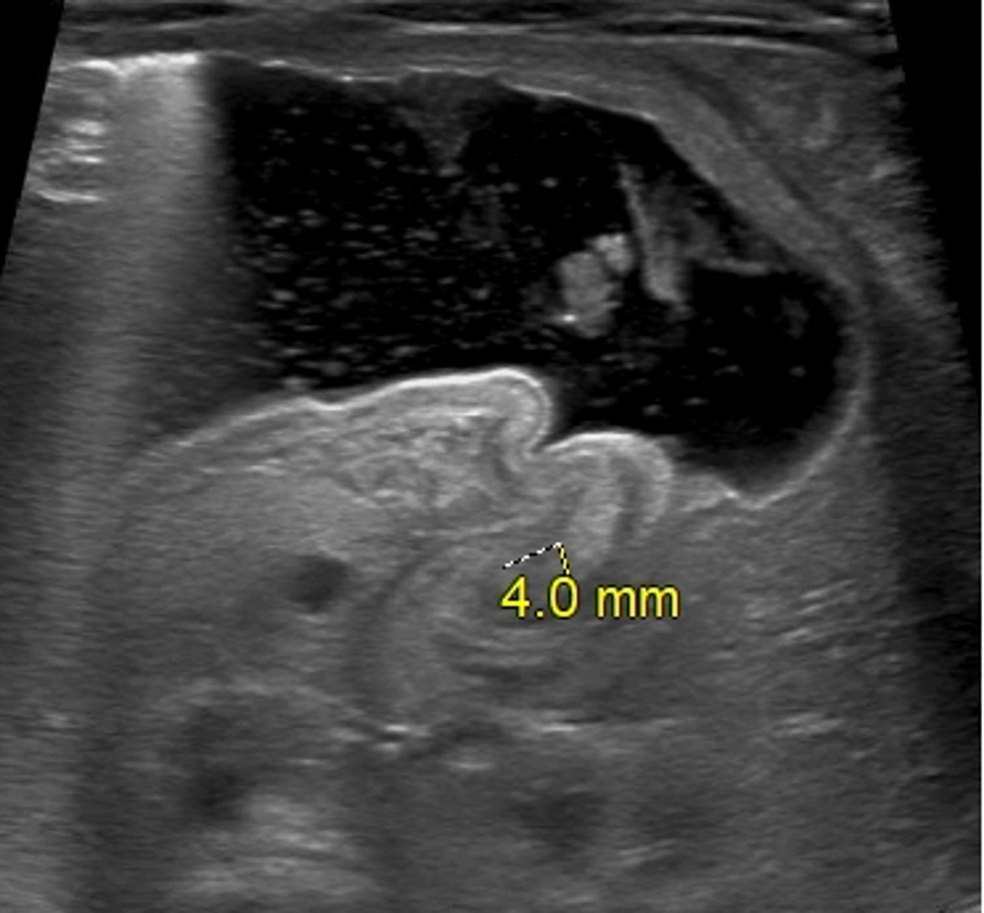
Transabdominal ultrasound revealing pyloric channel thickness measuring 4 mm as represented by yellow dotted line.

Biochemistry revealed Na+ 135mmol/L, K 6.2mmol/L, Cl 97mmol/L, HCO3 25mmol/L, glucose 94mg/dL, blood urea nitrogen (BUN) 6mg/dL, Ca++ 11mg/dL and anion gap of 13mmol/L. Complete blood count including hemograms were within the normal range with an inability to perform platelet count, which on repeat evaluation was 458x103/mcl. An abdominal x-ray was performed which showed evidence of gaseous distention of the stomach and ultrasound of the abdomen revealed thickening and elongation of the pyloric channel with a pyloric channel measuring 4 mm (Figure *1*). COVID-19 polymerase chain reaction (PCR) was performed with concerns for active COVID-19 pandemic during the time and the test was negative.

Twin B

**Figure 2:**
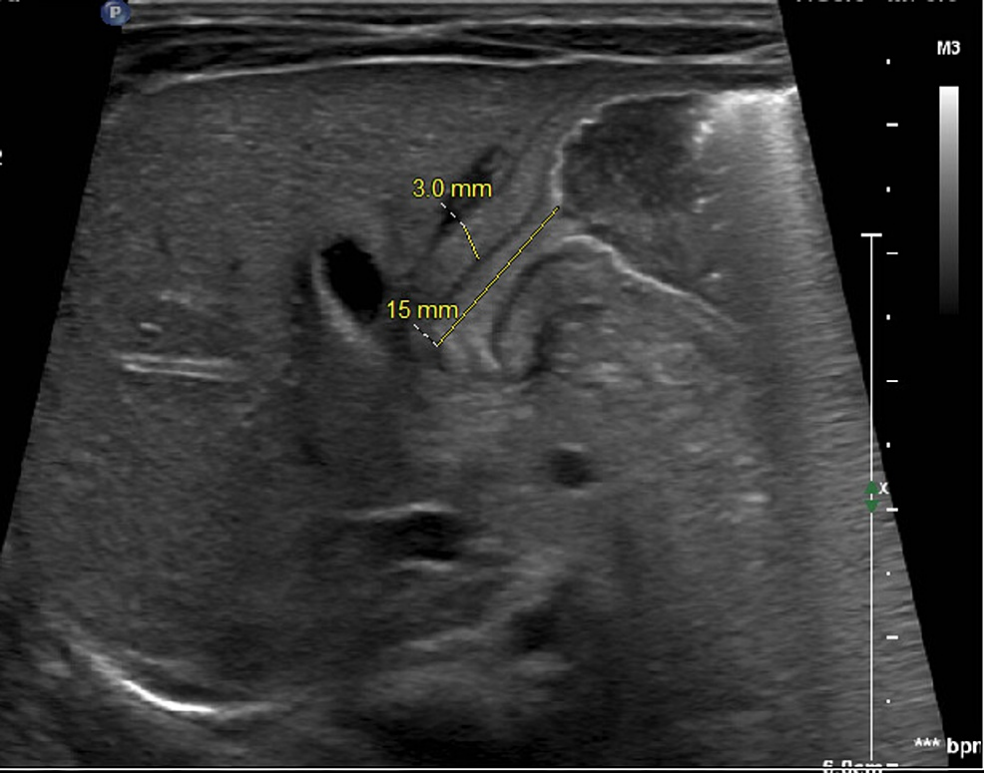
Transabdominal ultrasound demonstrating pyloric wall thickening of 3 mm and elongation of the pylorus 15 mm as represented by dotted yellow lines.

Biochemistry revealed Na+ 146mmol/L, K+ 7.4mmol/L, Cl 116mmol/L, HCO3 19mmol/L, glucose 83mg/dL, BUN 6, Ca++ 10.1mg/dL and anion gap of 11mmol/L. Complete blood count including hemogram was within normal limit. The abdominal ultrasound was performed which showed wall thickening of 3 mm and elongation of the pylorus 15mm and little to no gastric content progressing through the channel (Figure *2*). No contrast study was performed on this patient because of strong clinical suspicion and ultrasonography findings.

Treatment

After strong clinical suspicion, investigation, and radiologic finding on both infants, diagnosis of hypertrophic pyloric stenosis was made. Following initial preoperative stabilization and maintenance of intravenous hydration, both infants underwent laparoscopic pyloromyotomy. Feeding was reintroduced six hours postoperatively and gradually increased in amount every three hours until the next 24 hours with a plan to reintroduce the same amount in three hours if vomiting occurred. Both infants tolerated the procedure well and had no vomiting during the postoperative hospital stay.

Outcome and follow-up

Both infants were followed up in the primary care clinic postoperatively with adequate weight gain, growth, and development appropriate for age. This condition could be due to genetic predisposition but no further genetic analysis was performed in these infants and no history of IHPS was present in either the maternal or paternal side of the family. Environmental factors like formula feeding which have previously been attributed as one of the risk factors were present in both infants preceding the occurrence of symptoms.

## Discussion

IHPS is one of the most common causes of vomiting in infancy. Usually, infants with IHPS present within four to six weeks of life with clinically significant dehydration following the gradual onset of non-bloody, non-bilious vomiting [3]. Typical presentation could be a dehydrated hungry infant, feeding with repeated vomiting, and progressive signs of dehydration with the decrease in the frequency and amount of stool and urine. Though electrolyte imbalances are common, the significant signs and symptoms occurring due to electrolyte imbalance are less likely to be the presenting signs, as the infant is clinically worse and mostly rushed to hospital before these signs could appear. However, with the rising awareness among the parents and available resources for self-education infants may present even before the appearance of the signs of dehydration like in the case of Twin A, who presented at two weeks of life with projectile vomiting hemodynamically stable and not in dehydration. Radiologically thickening of the pyloric muscle and the elongation of the pyloric channel are the most significant findings with the muscle considered to be hypertrophied with a thickness of more or equal to 3mm [4].

Some features related to IHPS with regards to previous literature points towards hereditary patterns like male preponderance and increased occurrence among monozygotic twins and similar occurrence in dizygotic twins [5]. However, no specific autosomal or recessive pattern of inheritance has ever been observed in the occurrence of IHPS.

Many studies have tried to relate a genetic association for IHPS identifying multiple genetic loci among the affected members of the same family which could have a relation to IHPS. Serra et al. investigated neuronal nitric oxide synthase (NOS1) as a candidate gene as it is related to the smooth muscle relaxation and one of its variant gene’s ‘-84g>a’ association with IHPS. This study was able to identify 19 different polymorphisms in the coding region for the NOS1 gene with a statistically significant association but was unable to show the significance after Bonferroni adjustment for multiple testing [6].

Another study, Capon et al., conducted to identify the linkage of monogenic IHPS to chromosome 16p12-p13, performed mapping of the first locus for monogenic IHPS through multigeneration pedigree but concluded that chromosome 16 is unlikely to be the common locus for IHPS [7].

## Conclusions

Though genetic factors may contribute to the occurrence of IHPS, environmental factors demonstrate a strong association for the condition to occur. It can be justified by the difference in the appearance of symptoms, duration of onset, and the feeding habits among different infants with IHPS.

The etiopathogenesis of IHPS is yet to be completely explored despite multiple studies and currently, it is still believed to have a poly-etiologic origin including genetic as well as environmental factors.
